# Constitutional Mismatch Repair Deficiency Syndrome in a patient from India

**DOI:** 10.1002/ccr3.3249

**Published:** 2020-09-03

**Authors:** Chandramallika Paul, Subhosmito Chakraborty, Sarit Chakraborty, Kalyan Goswami

**Affiliations:** ^1^ Department of Biochemistry All India Institute of Medical Sciences (AIIMS‐Kalyani) Kalyani India; ^2^ Department of Clinical Biochemistry TATA Medical Center Kolkata India; ^3^ Department of Computer Science Engineering Government College of Engineering & Leather Technology Kolkata India

**Keywords:** constitutional mismatch repair deficiency, electrophoresis, microsatellite instability

## Abstract

This report highlights an extremely rare genetic condition constitutional mismatch repair deficiency (CMMRD) in an Indian pediatric patient with dual malignancies, who suffered from transient encephalopathy, a rare side effect of the drug Nivolumab and the associated challenge during CSF protein electrophoresis interpretation.

## Graphical abstract

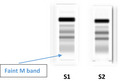

## 1 INTRODUCTION

This is a first case report of a patient with constitutional mismatch repair deficiency (CMMRD) from India who was found to suffer from encephalopathy after treatment with monoclonal antibody, Nivolumab, a check point inhibitor, based on monoclonal band in CSF protein electrophoresis (CSF‐PEP) while no band in serum protein electrophoresis (SPEP) or CSF‐immunofixation (CSF‐IFE).

CMMRD is an autosomal recessive disorder that occurs as a result of homozygous or compound heterozygous biallelic deleterious germline mutations in one of the four well‐characterized mismatch repair (MMR) genes including MLH1, MSH2, MSH6, and PMS2. The patients suffer from a broad spectrum of aggressive and pediatric cancers, like hematological malignancies, brain tumors, and colorectal cancers. In contrast to CMMRD, Lynch Syndrome is an autosomal dominant heterozygous monoallelic germline loss‐of‐function mutations in one of the four MMR genes with an increased risk of colorectal cancer, endometrial carcinoma, and other malignancies in the fourth and fifth decades of life.^1^, ^2^ The CMMRD patients usually have very short life span (mostly before adulthood); thus, prompt diagnosis and early intervention are extremely important.

Confirmation of the diagnosis involves the analysis of microsatellite instability panel (MSI) and/or immunohistochemistry (IHC) followed by mutation analysis.^3^ Current CMMRD treatment is based on immune modulators like checkpoint inhibitors, which counteract the actions of proteins that impede the immune response to cancer. Blocking PD‐1(programmed death) in CMMRD tumors ultimately allows activation of T cells which kills the cancer cells. CMMRD tumors are more responsive to PD‐1 blockers like Nivolumab than MMR proficient tumors as shown in recent literature.^4^

We herein report a case with CMMRD with childhood colorectal cancer and brain tumor, GBM (glioblastoma multiforme). He received adjuvant treatment for GBM with Nivolumab, a monoclonal antibody but showed signs of suspected immune encephalopathy post‐Nivolumab, an extremely rare complication of Nivolumab.^5^, ^6^ On biochemical evaluation, the CSF PEP showed an unusual and unexplained faint monoclonal M band near beta region without any type of restriction in serum protein electrophoresis (SPEP) or CSF‐IFE.

We would like to present this case because of its rarity and severity, rare side effect like encephalopathy with new immune modulators like Nivolumab, and its unusual electrophoretic interference.

The study received approval from the local ethics committee.

## 2 CASE REPORT

The patient is a 11‐year‐old boy from Jharkhand, India, who presented with pain abdomen and intermittent constipation two years back. Colonoscopy revealed polypoid stricturing lesion suggestive of sigmoid colon tumor which was further confirmed by radiological findings on CT (Computed Tomography) scan abdomen. Among nonmalignant features, dermatological feature in the form of hypopigmented macule on left shoulder was present. On reviewing the family history, there was a history of neural tube defect and early death in sibling, and a history of sudden death in paternal cousin at 14 years of age. Few incidences of consanguineous marriage were reported in their extended family. The patient underwent exploratory laparotomy and resection of polypoid segment of sigmoid colon. Histopathological examination (HPE) revealed moderately differentiated adenocarcinoma of the sigmoid colon (pT3N0). The MMR IHC done on colonic tumor block showed intact nuclear staining with MSH2, MSH6, and MLH1 while immunostain for PMS2 was attempted thrice but cannot be reported as the results were noncontributory. In view of the absence of PMS2 in both the tumor tissue and the native normal tissue, a possibility of constitutional MMR deficiency (CMMRD) was considered. The patient was planned for chemotherapy with Capecitabine. After 2 months of chemotherapy, he again presented with episodes of headache, vomiting with left‐sided facial weakness of supranuclear type and difficulty walking. CECT (Contrast‐Enhanced Computed Tomography) scan of the brain revealed right frontoparietal space‐occupying lesion (SOL) with perilesional edema and midline shift suggestive of brain tumor (glioma) for which he underwent craniotomy and surgical removal of the tumor. CNS panel on biopsy diagnosed glioblastoma multiforme (GBM) WHO grade IV. Microscopic examination revealed a proliferating glial tumor with marked nuclear pleomorphism, mitosis, necrosis, and microvascular proliferation. IHC showed tumor cells to be positive for GFAP (focal, strong), IDH1(R‐132H) was negative signifying a poorer outcome, ATRX was retained, high Ki67 index of around 70% signifying higher tumor recurrence and progression. IHC done for mismatch repair (MMR) proteins on the block of GBM showed intact nuclear staining for MLH1 and loss of nuclear expression for PMS2 in both the tumor cells and the normal brain tissue. Because of the absence of PMS2 in the tumor tissue, native normal tissue of both GBM block and prior colonic block, the diagnosis of constitutional MMR deficiency (cMMRD) was established. Mutation analysis subsequently confirmed the diagnosis. The patient had a disease‐free survival for next 3 months after which he again showed signs of progressive disease He was then further managed by salvage immunotherapy with Nivolumab, a monoclonal antibody. After 2 courses of Nivolumab given 1 month apart, the patient had episodes of vomiting, but not associated with headache or other clinical signs of raised intracranial pressure. As a part of management, Ommaya shunt was done with drainage of approximately 7.5 mL clear CSF (cerebrospinal fluid). CSF routine examination showed cell counts within normal limits and absence of any malignant cell while on biochemical evaluation CSF glucose was within normal limit but protein concentration was highly elevated to 563 mg/dL, the reference range being 12‐60 mg/dL. No metabolic abnormalities or acute infections or disease progression was evident from pathological, or microbiological work up. Contrast‐enhanced MRI (Magnetic resonance imaging) brain revealed right frontal lobe enhancing lesion and significant cerebral edema. CSF electrophoresis was done to rule out any inflammatory or noninflammatory neurological disease. In CSF electrophoresis (CSF‐PEP), an unusual faint M band near the beta region was seen, while in SPEP and CSF‐IFE, no heavy or light chain restriction pattern was noted corresponding to the M band as shown in Figure S1, S2, and S3, respectively. The patient was therefore diagnosed with immune encephalopathy post‐Nivolumab therapy. He was further treated with intravenous dexamethasone. The patient became stable and was discharged after 2 days on a dexamethasone taper. Considering the benefits of significant tumor reduction in contrast to the rare side effect malignant cerebral edema as reported by ^5^, the patient was further managed by 6 courses of salvage Nivolumab therapy for next 6 months. Significant reduction of tumor lesions was evident from subsequent brain MRI without any repeat episode of nivolumab induced cerebral edema. However, despite 6 months of salvage immunotherapy, the patient suffered from progressive disease owing to the severity of glioblastoma and CMMRD disease spectrum. The patient was managed henceforth by only palliative therapy. Age at last follow‐up of the patient was 11 years.

## 3 DISCUSSION

The patient was diagnosed and treated for CMMRD at Tata Medical Center, Kolkata, India. The characteristics are evaluated and presented in supporting information Table S1. Age at diagnosis is consistent with the available literature on CMMRD.^2^ The diagnosis was established by immunohistochemical stain (IHC) with lack of PMS2 staining suggestive of microsatellite instability and subsequently confirmed by molecular genetic testing. The patient suffered from the classical CMMRD tumor spectrum involving colorectal cancer and glioblastoma. The patient underwent surgical and medical management with radiotherapy. Check point inhibitor Nivolumab was used as salvage therapy for GBM. Nivolumab is a monoclonal antibody that acts as an immune checkpoint by blocking programmed cell death protein 1 and activating the immune system against cancer cells. It is noteworthy that the patient experienced signs of transient immune encephalopathy, an extremely rare side effect of Nivolumab therapy.^5^, ^6^ During the biochemical evaluation, CSF and serum protein electrophoresis results showed an unusual correlation in the form of a faint M band near the beta region in CSF‐PEP while in SPEP/CSF‐IFE, no corresponding heavy or light chain restriction pattern was noted. Usually, the detection of multiple bands or oligoclonality in CSF study is a sign of intrathecal immunoglobulin synthesis indicating a variety of immunological disorders mostly multiple sclerosis. Very few studies^7^, ^8^ have addressed the issue of this type of unusual solitary bands in CSF protein electrophoresis especially in cases of drug‐induced immune encephalopathy.

In a study done by^7^ six classic electrophoretic patterns were observed from a wide patient group mostly involving multiple sclerosis (Table S2). Types 2 and 3 indicated intrathecal synthesis, and types 1, 4, 5, and 6 were considered as negative results. Since cerebrospinal fluid (CSF) is an ultrafiltrate of plasma, it has much lower concentrations of the highest molecular weight proteins such as IgG, IgA, and IgM.

In conclusion, this case report justifies the need for CMMRD reports in a developing country like India and a better understanding of the associated challenges in electrophoresis with emerging therapeutics like Nivolumab.

## CONFLICT OF INTEREST

None declared.

## AUTHOR CONTRIBUTIONS

All authors: involved in the conception, design, write‐up, and revision of this article. CP_1_ and SC_2:_ planned the study. CP_1:_ collected the data and wrote the report. SC_3_ and KG_1:_ analyzed, edited, and revised the manuscript.

## 4 ETHICS APPROVAL

This study was approved by the institutional ethical committee of the TATA MEDICAL CENTER, KOLKATA, INDIA. All personal identifiers (name, employer, and contact) were removed from the data set, and analyses were carried out at the institution level.

## Supporting information

Figures S1‐S3

Tables S1‐S2

## References

[1] Wimmer K , Kratz CP .Constitutional mismatch repair‐deficiency syndrome. Haematologica. 2010;95(5):699‐701. 20442441 10.3324/haematol.2009.021626 PMC2864372

[2] Lavoine N , Colas C , Muleris M , et al. Constitutional mismatch repair deficiency syndrome: clinical description in a French cohort. J Med Genet. 2015;52(11):770‐778. 26318770 10.1136/jmedgenet-2015-103299

[3] Wimmer K , et al. Diagnostic criteria for constitutional mismatch repair deficiency syndrome: suggestions of the European consortium ‘Care for CMMRD’ (C4CMMRD)/. J Med Genet. 2014;51(6):355‐365. 24737826 10.1136/jmedgenet-2014-102284

[4] Abedalthagafi M . Constitutional mismatch repair‐deficiency: current problems and emerging therapeutic strategies. Oncotarget. 2018;9(83):35458‐35469. 30459937 10.18632/oncotarget.26249 PMC6226037

[5] Zhu X , McDowell MM , Newman WC , Mason GE , Greene S , Tamber MS . Severe cerebral edema following nivolumab treatment for pediatric glioblastoma: case report. J Neurosurg Pediatr. 2017;19(2):249‐253. 27858578 10.3171/2016.8.PEDS16326

[6] Leitinger M , Varosanec MV , Pikija S , et al. Fatal Necrotizing Encephalopathy after Treatment with Nivolumab for Squamous Non‐Small Cell Lung Cancer: Case Report and Review of the Literature. Front Immunol. 2018;9:108. 29441072 10.3389/fimmu.2018.00108 PMC5797606

[7] Pinar A , Kurne AT , Lay I , et al. Cerebrospinal fluid oligoclonal banding patterns and intrathecal immunoglobulin synthesis: Data comparison from a wide patient group. Neurol Sci Neurophysiol. 2018;35(1):21‐28.

[8] Hegen H , Zinganell A , Auer M , Deisenhammer F . The clinical significance of single or double bands in cerebrospinal fluid isoelectric focusing. A retrospective study and systematic review. PLoS ONE. 2019;14(4):e0215410. 30986255 10.1371/journal.pone.0215410 PMC6464233

