# Supplementary material for: Constitutional Mismatch Repair Deficiency Syndrome in a patient from India
Source: Clin Case Rep. 2020 Sep 3;8(12):2824–6. doi: 10.1002/ccr3.3249 (PMC7752390; doi:10.1002/ccr3.3249)
Supplement: Supplementary file 1 — Figures S1‐S3 [file CCR3-8-2824-s001.docx]

**** ****
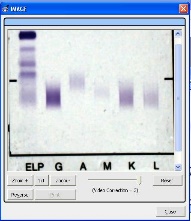


**Faint M band**

**S1**  **S2 S3**

**FIGURE: (S1)** CSF PEP: 1 faint but sharp M band near beta region **FIGURE:** **(S2)** no M- band in SPEP **FIGURE:** **(S3)** CSF IFE: no heavy or light chain restriction pattern
