# Supplementary material for: Constitutional Mismatch Repair Deficiency Syndrome in a patient from India
Source: Clin Case Rep. 2020 Sep 3;8(12):2824–6. doi: 10.1002/ccr3.3249 (PMC7752390; doi:10.1002/ccr3.3249)
Supplement: Supplementary file 2 — Tables S1‐S2 [file CCR3-8-2824-s002.docx]

**Supporting information**

**Table S1** Characteristic of the Proband

| Gene | PMS2 |
| --- | --- |
| Family history | Early death in sibling and paternal cousin |
| Score | 8 |
| Malignant tumors/polyps (age in years at diagnosis) | CRC/polyps(9)  Gliobastoma(10) |
| Cutaneous signs | hypopigmentation |
| IHC | PMS2- |
| Outcome(age in years at last follow-up) | Tumour progression(11) |

(CRC, colorectal cancer; Score, score according to Wimmer’s criteria *[Katharina Wimmer et al., 2014]* at diagnosis of first malignancy; IHC, immunohistochemistry)

**Table S2** CSF electrophoretic analysis

| Type | CSF Bands | SPEP bands |
| --- | --- | --- |
| 1 | **Nil** | **Nil** |
| 2 | **Oligoclonal IgG** | **Nil** |
| 3 | **Oligoclonal + additional bands** | **Oligoclonal** |
| 4 | **Oligoclonal** | **Oligoclonal(identical to CSF)** |
| 5 | **Monoclonal** | **Monoclonal** |
| 6 | **Single band** | **nil** |

(Type 2 and 3 indicate intrathecal synthesis, Type 1, 4, 5, and 6 are considered as negative results)
